# An Assessment and Analysis Model of Psychological Health of College Students Based on Convolutional Neural Networks

**DOI:** 10.1155/2022/7586918

**Published:** 2022-06-22

**Authors:** Panpan Li, Feng Liang

**Affiliations:** College of Healthy Management, Shangluo University, Shangluo, Shaanxi 726000, China

## Abstract

Psychological health assessment and psychological problem identification essentially belong to problems of pattern recognition or nonlinear classification; its system contains complex nonlinear interactions among various factors, having basic characteristics of multivariable, multilevel, and strong coupling. An important problem in the field of artificial intelligence solved by convolutional neural networks (CNN) is to simplify complex problems, minimize the number of parameters, and thus greatly improve the algorithm's performance. Therefore, CNN has outstanding advantages in establishing the assessment and analysis model of college students' psychological health. This study determined the psychological health standards of college students, selected measurement tools for college students' psychological state, elaborated the principles of psychological assessment based on text information, performed the sample set data establishment and data processing of the assessment and analysis model of psychological health, conducted network establishment, training, and simulation, carried out a case experiment and its result analysis, explored the cause analysis of college students' psychological health problems, and finally discussed the prevention and intervention of college students' psychological problems. The study results show that the input and output of the CNN-based assessment and analysis model of college students' psychological health are their evaluation data and assessment results, respectively, and the optimal hyperparameters of the model are determined through fold cross-validation analysis to improve the model's over-fitting problem. After the training is completed, the model can predict the changes in college students' psychological state in the future through the psychological test data. The CNN uses supervised machine learning method to construct an assessment and analysis model of college students' psychological health, and establishes the mapping relationship between college students' personal background and their psychological health. The network error continuously adjusts network connection weight according to gradient descent algorithm to minimize its error, so that the convolutional layer and the pooling layer can learn the optimized feature expression of the input data.

## 1. Introduction

Psychological health is a harmonious state in which individual psychological functions are in balance and the most common abnormal states of psychological health are anxiety and depression. College students are at the transition stage between campus and society, facing pressure from all sides, and are prone to various psychological problems, among which depression is particularly prominent [1]. The scientific and perfect assessment and analysis model of college students' psychological health is of great significance for evaluating whether college students are psychologically healthy or not, promoting college students' psychological health education, and ultimately realizing the healthy development of college students' psychology [2]. On the one hand, the part of speech is integrated into the word vector as the input of the text, which plays the role of word sense disambiguation and improves the quality of the neural network input. On the other hand, considering the structural information of the text, the model adopts a segmentation pooling strategy to extract the maximum features of sentences. However, the formulation of the current college students' psychological health assessment and analysis model lacks scientific and unified standards, and the selection of assessment indicators is too single. However, due to the variability, urgency, uncertainty, complexity, multiplicity, and contingency of college students' psychological health problems, psychological health assessment has become a more difficult subject [3]. In this regard, it is necessary to carefully study the psychological characteristics of contemporary college students on the basis of analyzing the factors that affect the psychological health of college students, and establish a professional analysis model for the assessment of college students' psychological health [4].

Convolutional neural network (CNN) has significant advantages in mental health assessment of college students. The training algorithm based on the CNN effectively solves the problem of gradient disappearance in mental health analysis, which makes the effective time of training longer. The mental health assessment and analysis of college students requires model support with high generalization, and the CNN algorithm with high complexity and capacity has good generalization in the big data environment [5]. The CNN can help mental health analysis extract more and more effective information from massive data and it also has the features of layer-by-layer construction, which can extract higher-level features in psychological assessment, and decompose the interacting influencing factors into independent and more effective factors [6]. Evaluation of the number of mapping relationships in the model can predict the pros and cons of single-layer and multi-layer model accuracy; indicators can be aggregated into layers or element groups with basically the same attributes, and there is no mutual influence or dominance between elements within the layers. The CNN adds a feature learning part on the basis of the traditional multi-layer neural network, and uses the spatial relative relationship to reduce the number of parameters to improve the training performance of college students' mental health assessment and analysis [7]. The CNN is to simplify complex problems and minimize the number of parameters, thereby greatly improving the performance of the algorithm [8].

On the basis of summarizing and analyzing the previous research works, this study will expound the research status and significance of college students' psychological health assessment, elaborate the development background, current status and future challenges of the CNN, determine psychological health standards of college students, select measurement tools for college students' psychological state, elaborate the principles of psychological assessment based on text information, perform the sample set data establishment and data processing of the assessment and analysis model of psychological health, conduct network establishment, training and simulation, carry out a case experiment and its result analysis, explore the cause analysis of college students' psychological health problems, and finally discuss the prevention and intervention of college students' psychological health problems. The results of this paper are expected to provide a reference for further research on the assessment and analysis model of college students' psychological health based on convolutional neural networks. The detailed chapters are arranged as follows: Section 2 introduces research methods and principles; Section 3 establishes the assessment and analysis model of college students' psychological health based on the CNN; Section 4 carries out a case analysis and its result discussion; Section 5 is conclusion.

## 2. Research Methods and Principles

### 2.1. Determination of Psychological Health Standards for College Students

The previous psychological health assessment method measures the symptom level of a certain period of time, which reflects a person's psychological good or bad state in a certain period of time at that time, and is easily affected by many factors, especially the influence of life events, such as it is normal for college students facing important exams to have high anxiety scores. At the first-order level, this study uses 16 factors of the psychological health structure of college students: persistence and experiential learning, helpfulness and cooperation, fulfillment and responsibility, direction and purpose, urgency and competence, self-regulation and confrontation reality, purposefulness, emotional control and self-acceptance, independence, interpersonal perception, self-adaptation, development and perfection, self-motivation and future orientation, empathy and trust, self-awareness and cooperation, initiative and sensitivity. At the second-order level, this study obtained a 5-factor structural model: individual growth, positive relationships with others, autonomy, environ-psychological control and utilization, and life goals [9]. Adding relevant indicators for evaluating psychological health or increasing their corresponding proportions in the assessment index system of college students' psychological health is not only in line with the psychological health standards of various schools of psychology, but also an urgent need to solve the psychological crisis of contemporary college students.

### 2.2. Selection of Psychological Measurement Tools for College Students

The psychological state of each independent individual is a multi-dimensional information system, and its system contains complex nonlinear interactions among various factors, having basic characteristics of multivariable, multilevel, and strong coupling, which is to be described by traditional mathematical methods. The CNN prediction model can predict the psychological health score of college students by calculating the information of various factors that affect the psychological health state of college students. The predicted psychological health score of college students is not final, but the psychological pressure caused by the influence of various factors on college students and this predicted value is used as a guiding basis for psychological counseling work [10]. If the predicted value exceeds a certain value, it means that the students have greater psychological pressure and need to carry out later psychological interventions. The model uses psychological counseling, heart-to-heart, and other means to understand which factors are mainly causing the pressure, and then it adjusts the students' psychology in time, and then returns to the normal level. They need to be converted into numerical features that can be recognized by machine learning algorithms, and then handed over to machine learning algorithms for operation. The systematic principle requires the model to start from a systematic perspective, comprehensively use qualitative knowledge such as psychology, and comprehensively and systematically find out the factors that affect the students' psychological health.

### 2.3. Psychological State Assessment Based on Text Information

**Figure 1:**
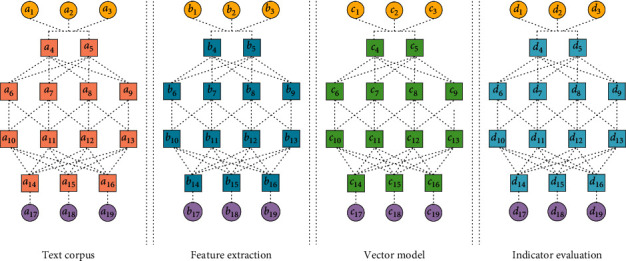
Psychological state assessment based on text information in the assessment and analysis model of psychological health of college students.

The accuracy of the optimization categories of the mental health assessment analysis model of college students needs to be evaluated through the corresponding evaluation system. With the increase of training, the complexity increases, and it is prone to over-fitting; in the evaluation process, the input multivariate data is analyzed to extract the necessary psychological state. In addition, hidden association rules not discovered by classification can also be discovered based on the correlation of mental states, which provides knowledge for further segmentation of mental states. The systematic principle of assessment and analysis of college students' psychological health requires the model to start from a systematic perspective, comprehensively use qualitative knowledge such as psychology, and comprehensively and systematically find out the factors that affect students' psychological health. The model uses quantitative methods such as fuzzy mathematics to comprehensively evaluate the impact indicators; the principle requires that each indicator to be evaluated must have a clear connotation and scientific explanation. By extracting the mental states at the significance level, the accuracy of the analysis is improved, and the amount of computation and memory usage is reduced, and common mental states can be discovered. In the second assessment, the correlation between individual mental states and common mental states was identified, and through this process, positive and negative correlations between mental states were found. As the training increases and the accuracy improve, the memory capacity and computation speed also need to increase and some high-precision layers should be used according to the purpose of data analysis [11].  Figure 1 shows psychological assessment based on text information in the model of college students' psychological health.

The CNN uses the sliding window technology to convert the usage data in the form of time series into a series of data units, and on this basis, establishes a CNN structure suitable for the assessment and analysis model of college students' psychological health. The input of the model is the psychological health data of college students, and the output is the results of the psychological health assessment of college students. The optimal hyper-parameters of the model are determined by fold cross-validation analysis and the over-fitting problem of the model is improved. The CNN uses supervised machine learning method to construct an assessment and analysis model of college students' psychological health, and establishes the mapping relationship between college students' personal background and psychological health. The infiltration of various intervention methods is to make the intervention work of college students' psychological disorder reflected in the daily teaching and management of colleges and universities. The network error continuously adjusts the network connection weight according to the gradient descent algorithm to minimize the error, so that the evaluation stage and the analysis stage can learn the optimal feature representation of the input data. The construction of the model is mainly divided into two stages: model evaluation stage and model prediction stage. The model inputs the data processed by the sliding window into the type optimization module; the type optimization is realized by alternately cascading the evaluation phase and the analysis phase; these operations are performed on the data unit, and finally the features learned from various signal sources are combined as an evaluation and analysis input.

## 3. Establishment of CNN-Based Assessment and Analysis Model of College Students' Psychological Health

### 3.1. Data Establishment and Data Processing of Sample Set

**Figure 2:**
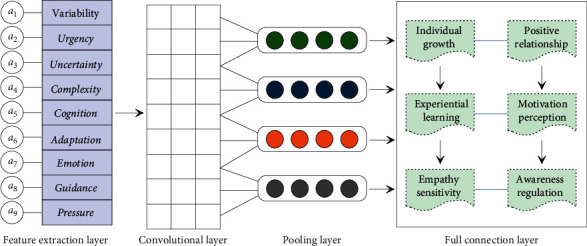
Data establishment and data processing of sample set in the CNN-based assessment and analysis model of college students' psychological health.

The assessment and analysis model of college students' psychological health needs a refined analysis of the stimulating effect of environ-psychological factors. Environ-psychological factors refer to factors that directly or indirectly affect individual psychology in the spiritual and material environments. Psychological environment includes micro-psychological environment and macro-psychological environment. The micro-psychological environment refers to the social relationship circle that has a direct impact on the psychology and has a large stimulating effect, and has the most direct impact on the personality development, temperament shaping, cognition, and emotional regulation of college students. As shown in Figure 2, relatively intimate psychological factors such as peer relationships, social culture, campus culture, network culture, natural environment, and other factors constitute the macro-psychological environment of college students' psychological health. In addition, environ-psychological factors can also be divided into normal environment and mutation environment. The normal environment is a long-term relatively stable objective environment, and the individual psychology grows and changes gradually in the normal environment; the mutation environment is caused by unpredictable situations such as intense emotional stimulation, major diseases, major changes, major accidents, and natural disasters. The objective environment caused by emergencies has a large psychological impact, and it is easy to cause psychological trauma and even psychological distortion.

The types of college students' psychological disorders include many aspects, including cognitive problems, adaptation problems, and emotional problems. There are both pressure problems and orientation problems. The psychological problems of college students are social on the basis of biology. Standardization means that the test conditions are basically the same, and the same evaluation questions and unified standards are used for interpretation and evaluation. However, for subjects with different abilities and different characteristics, it is impossible to truly measure their real situation. According to the classic evaluation, the various statistical values obtained by theory are overly dependent on samples and the mental health referred to in these assessments is the mental health based on commonality. Those behaviors or performances that do not conform to the routine will be rated as biased, which is obviously unjust, because what does not conform to the routine is not necessarily wrong. Another method is to extract the principal components of the index features through the principal component analysis method, complete the processing of the index features, and finally realize the division of the test set and the training set [12]. The multivariate analysis of variance tests whether the mean vectors of multiple populations in the mental health assessment of college students are equal. It mainly judges whether the mean vectors of multiple populations are equal by analyzing the error source of each observation data. The data comes from random sampling, and the observations in the sample are independent of each other.

Because liberal arts students often involve interdisciplinary subjects related to psychology, and non-medical science students have less contact, the knowledge level of science students' mental health literacy is relatively low, which also reflects the shortcomings of the mental health service system for college students. With the continuous development of the social economy and the increasing employment pressure, it is more and more difficult for college students to find their favorite jobs after graduation, which leads to psychological problems such as anxiety and paranoia. A good social system and proper relaxation can relieve the pressure brought by the imminent employment of college students and improve their mental health. Interpersonal communication is the starting point of socialization. Positive interpersonal communication helps college students obtain richer information and maintain contact with society; positive and harmonious interpersonal relationships also help college students develop good personalities [13]. The system of college students' mental health assessment and analysis and the principle of sexuality require the model to comprehensively and systematically find out the factors that affect the mental health of students from a systematic perspective, comprehensively using qualitative knowledge such as psychology and pedagogy. The network used in this model is an optimized mode that uses local receptive fields to perform function mapping based on the knowledge of biological local adjustment and overlapping receptive regions.

### 3.2. Network Establishment, Training, and Simulation

**Figure 3:**
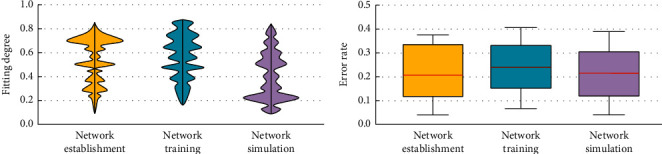
Network establishment, training, and simulation in the CNN-based assessment and analysis model of college students' psychological health.

There are various reasons for college students' psychological disorders, which may be the reasons of schools, families, or students' personal reasons; there are problems caused by students' personality distortion and cognitive impairment, and there are also consequences caused by poor school management and education. The types of college students' psychological disorders include many aspects, including cognitive problems, adaptation problems, and emotional problems. There are both pressure problems and orientation problems. The psychological problems of college students are social on the basis of biology. Therefore, to carry out targeted education for different groups and different categories of psychological problems, the intervention of college students' psychological disorders needs to be based on the existing resources of colleges and universities, and the existing conditions to tap the potential [14]. The multi-dimensional systematization of intervention methods is to mobilize all resources and carry out intervention work from multiple channels and angles. After the training is completed, the model can predict the changes in the psychological state of college students in the future through the psychological health test data. By constructing a psychological health assessment system and intervention system for college students, the effectiveness and pertinence of college students' work can be enhanced, which can help to identify students' psychological problems in advance, take preventive measures in time, reduce management and education risks, reduce education costs, and enhance the psychology and comprehensive quality of college students (Figure 3).

In the actual psychological health assessment of college students, it is impossible to achieve enough assessment times or eliminate errors, so the concept of standardization is proposed to control and eliminate the assessment errors. Standardization means that the test conditions are basically the same, and the same assessment questions and unified standards are used for interpretation and assessment. However, for subjects with different abilities and different characteristics, it is impossible to truly measure their real situation. According to the classic assessment, the various statistical values obtained by theory are overly dependent on samples, which limit their generalization. This theory avoids the substantive question of what is the relationship between the internal characteristics of the subjects themselves and the tasks and external environments faced by the subjects. The latent trait theory includes three basic assumptions of ability latitude, local independence, and item characteristic curve, and concepts such as the distribution of ability and assessment scores, item information, and assessment information. This theory has the characteristics that the item and trait parameters are fixed, that is, the estimated value of the parameter is not affected by different samples. Even if the subject is assessed with two completely different item groups, the estimated subject's trait level will still be within the same range. Taking the value on the same measurement system can estimate the measurement error of different ability levels, which is very beneficial to the establishment of large-scale item banks and flexible assessment.

Taking the psychological survey of college students as an example, an effective standardized scale enables practitioners to obtain information on the psychological status of a large group in a relatively short period of time, and can identify individuals with special intervention needs according to the norm. These deviations are closely related and interact with each other, but it needs to be clear that there may be a common cause behind them. The common psychological problems of college students are mainly interpersonal communication, academic and employment pressure, and these two factors directly affect the deviations in emotion, behavior, and physiology at different college stages. In the study of psychological health standards, psychological health should have different levels and the psychological health referred to in these assessments is the psychological health based on commonality [15]. Those behaviors or performances that do not conform to the routine will be rated as biased, which is obviously unjust, because what does not conform to the routine is not necessarily wrong. Adaptation to the environment and achieving inner balance are one aspect and the continuous pursuit of growth and change to become a distinctive self is also another aspect. Therefore, the ideal psychological health assessment should be an assessment that takes into accounts both commonality and individuality, and an assessment that integrates the double standards of adaptation and development.

## 4. Case Analysis and Its Result Discussion

### 4.1. Sample Selection and Background Description

This study takes the students of a university in Guangzhou, Guangdong Province, southern China as an example, and conducts an empirical study on the assessment and analysis model of college students' psychological health based on the CNN. In this paper, the relevant indicators that affect psychological health status include family atmosphere, parenting style, family economic conditions, major family accidents, social transformation, values, social competition, employment difficulties, learning pressure, teacher education methods, school management system, school material conditions, majors studied, emotional problems, interpersonal relationships, physical and physiological diseases, family support, and personality. The assessment model based on the traditional method is to remove the characteristic variables by collinear processing of the characteristic variables, so as to obtain the preliminary screening variables. Then, on the one hand, the model is established according to the traditional assessment model construction method, and at the same time, two commonly used different feature processing methods are compared and analyzed to screen the feature variables: one method is to calculate the value of the information degree of the feature variable, and set a threshold according to its size. In order to further screen the feature variables; another method is to extract the principal components of the index features through the principal component analysis method, complete the processing of the index features, and finally realize the division of the test set and the training set (Table 1).

One-way multivariate analysis of variance is to consider multiple response variables as a whole when considering multiple corresponding variables in the psychological health assessment of college students, and find the largest between-group difference of different populations from any linear combination of response variables, that is, the overall effect of multiple response variables. The multivariate analysis of variance tests whether the mean vectors of multiple populations in the psychological health assessment of college students are equal. It mainly judges whether the mean vectors of multiple populations are equal by analyzing the error source of each observation data [16]. The joint distribution of the response variables is a multivariate normal distribution; the covariance matrix of each population is the same; there is a linear correlation between the response variables; the sample size of each group should be as large as possible, and the sample size of each group should be as large as possible. According to positive emotion theory, short-term focused solutions use various core skills to help clients improve their awareness of their lives and challenges, thereby further expanding the client's cognitive paradigm and behavior pattern, so as to address the issues that the client is concerned about. The core task of the short-term solution-focused therapist is not to change the client's cognitive or behavioral patterns from their own perspective, as is the case with cognitive behavioral therapy.

The average level of psychological health knowledge of college students is higher than the average level of residents, but there are large regional differences, environ-psychological differences, cultural differences, etc.; in terms of the knowledge level of psychological illness, the performance of college students is relatively poor. The overall understanding of psychological health knowledge among college students is relatively one-sided, and their knowledge is extremely limited; at the same time, the relatively low level of psychological health literacy knowledge also reflects the shortcomings of the psychological health service system for college students. A good social system and proper relaxation can relieve the pressure brought by the imminent employment of college students and improve their psychological health. Although students' minds have gradually matured, due to the uneven personality quality, cognitive biases are prone to occur, resulting in many students being unable to integrate into the university campus, which brings serious psychological burden and psychological pressure to college students. When college students identify their own psychological state, they are required to be able to grasp their own emotions and concepts at this moment, and they are required to be able to more correctly identify the emotional state of others, and to be able to find out when others are facing a crisis [17].

### 4.2. Assessment and Cause Analysis of College Students' Psychological Health Problems

**Figure 4:**
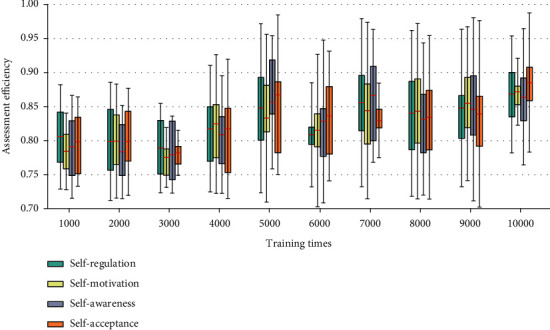
Assessment and cause analysis of college students' psychological health problems based on the CNN.

The college period is a very special and important stage and college students are transformed from the state of being taken care of to the state of independent living. During this process, various psychological problems are prone to occur. Family structure, family atmosphere, family education methods, family conditions, etc. will have a significant impact on students' psychology, and even cause students to form bad personalities. As shown in Figure 4, some family education methods and family structure will cause varying degrees of harm to students' psychological health. The knowledge level of psychological health literacy of college students in reading is one-sided knowledge system and vague knowledge content, and the difference between college students in different regions is obvious. The skill level of psychological health literacy includes various skills of psychological health promotion and psychological disease prevention mastered by individuals, such as identification of psychological state, help-seeking behavior, intervention, emotion regulation, and stress coping. With the continuous development of the social economy and the increasing employment pressure, it is more and more difficult for college students to find their favorite jobs after graduation, which leads to psychological problems such as anxiety and paranoia. If the situation continues for a long time, it will inevitably affect the psychological health of students, and even affect their outlook on life, values, and world outlook.

The transition from adolescence to an adult is a rather arduous and crisis-filled period. In addition to physical maturity and the accumulation and improvement of cultural knowledge and skills, what college students need to accomplish in the process of development is the establishment of personal roles and independence. They face not only their own internal growth and development, but also the tasks to be completed and various conflicts and challenges. Young people at the university stage begin to have a strong sense of self-fulfillment, self-development, and reinforcement. Therefore, in the assessment of the importance of psychological health for college students, the content about the self and the individual accounts for a considerable proportion. Interpersonal communication is the starting point of socialization. Active interpersonal communication helps college students obtain richer information and maintain contact with society; positive and harmonious interpersonal relationships also help college students develop good personalities [18]. Friendship, understanding and support, enhance self-confidence and sense of self-worth, relieve inner conflict and depression, and reduce loneliness and sense of loss. Therefore, establishing good interpersonal relationships with others has become the focus of college students' attention, and it has also become a measure indicator of psychological health with relevant studies empirically supported.

After word segmentation, text will form a set of words. For these word sets, machine learning algorithms cannot use them directly and they need to be converted into numerical features that can be recognized by machine learning algorithms, and then handed over to machine learning algorithms for operation in the transformation process, called feature extraction. Since the extraction of feature word vectors is based on the already trained word vector model, this study will train the word vector model, and then based on this word vector model, extract the feature word vectors from the previously segmented corpus [19]. The CNN has high complexity capacity and good generalization in the big data environment, and realizes the model support of college students' mental health assessment. The specific implementation method is to add a partially connected convolutional layer in front of the fully connected layer of the traditional neural network. The assessment model based on the traditional method is to remove the characteristic variables by collinear processing of the characteristic variables, so as to obtain the preliminary screening variables. The assessment indicators should be horizontally and vertically comparable and have the same dimension and indicators can be aggregated into hierarchies or element groups with basically the same attributes. There is no mutual influence or dominance between elements within the hierarchy, or this effect is so weak that it can be ignored.

### 4.3. Prevention and Intervention of College Students' Psychological Health Problems

**Figure 5:**
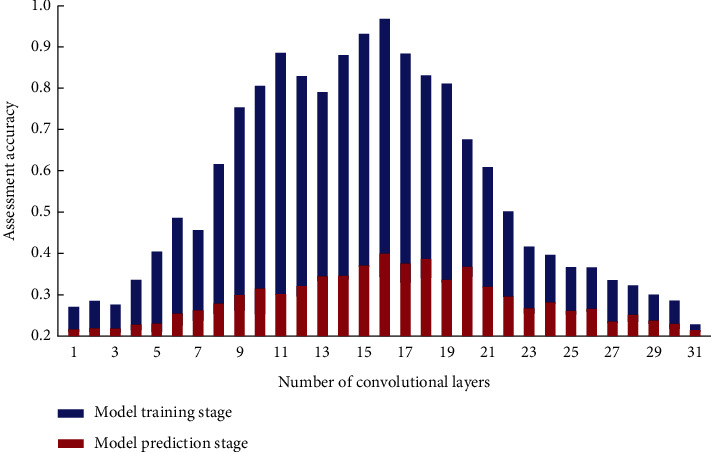
Relationship between Assessment accuracy of number of convolutional layer in the model training stage and prediction stage.

When determining the number of evaluations of the model, the number of manually annotated samples in the samples used in the convolutional neural network-based mental health evaluation analysis model for college students is small. If the evaluation is performed too many times, the CNN network will be over-fitted; if the evaluation is performed too few times, the accuracy of the model cannot meet the requirements. From the model accuracy of different evaluation times of the model under the analysis set and the optimization set, it can be seen that when the evaluation times of the analysis set are small, the accuracy consistency between the optimization set and the analysis set is higher, and the model accuracy is lower (Figure 5). When the number of times is large, the accuracy of the analysis set model will increase, but the gap between the accuracy of the optimization set and the analysis set model will become larger, and the model will be over-fitting at this time. The mental health assessment and analysis of college students is an artificial neural network that uses local receptive fields to perform function mapping based on the knowledge of biological local regulation and overlapping receptive regions [20]. The basic idea is to use the simulation module as the basis for the interpretation standard, to transform the input vector in the optimization stage of the psychological analysis module, and to transform the low-dimensional pattern input data into the high-dimensional mode, so that the problem of linear inseparability in the low-dimensional space is achieved with linearly separable in high-dimensional mode.

The core mechanism of interactive ritual theory is that by gathering in a given group of people, a common emotional experience can be stimulated, so that individuals can effectively integrate into new groups, thereby obtaining specific emotional energy groups. The interaction ritual chain theory of college students should refer to social interaction, that is, the process of ideological interaction and expression of feelings between college students and other people. Therefore, the possibility of establishing good relationships with others is also an important criterion for measuring the psychological health of college students. College students are in the transition stage from juvenile to adulthood. Students need to constantly learn and improve whether they are knowledge or social experience, and they need to have a correct understanding and assessment of themselves, so that their ideals and reality are consistent [21]. Although we advocate a sense of autonomy, this does not mean breaking away from the collective and reality. The meaning of self-care and autonomy is to put one's own efforts in the first place on the road to achieve ideals, rather than being divorced from the masses and reality. College students should learn to observe the environment, adapt to the environment, use the environment, and improve the environment, so as to maximize the space for personal development. The ones with life goals are often willing to try new things in life in pursuit of challenges and excitement, and can accomplish them well.

The assessment and analysis model of psychological health of college students reflects the state of psychological change through the fluctuation state of the three-dimensional dynamic curve. If the curve radian has a large drop at a certain inflection point, it means that the environ-psychological stimulation has a greater impact on the psychology, and the possibility and degree of psychological trauma are relatively large. If the stimulus of an event is very strong, the curve change is still within the normal threshold range or the gap is large but the duration is short, indicating that the individual has good psychological resilience, self-regulation, and resilience [22]. If the curve continues to fall for a long time, it means that the psychology has been in a state of inhibition, which is easy to induce psychological disorders or psychological diseases. The fluctuations of the psychological curve are all within the norm range, indicating that the psychological state is normal. They tend to be the standard of life adaptation, focusing on describing the common and obvious problems of college students' physical and psychological development, while ignoring the healthy development of college students on the spiritual level. In order to analyze the temporal performance of different neural network models under the same conditions, this paper uses the same word vector matrix for comparative experiments, and records the time it takes for different models to complete one iteration on the clothing review dataset.

## 5. Conclusions

This study determined psychological health standards of college students, selected measurement tools for college students' psychological state, elaborated the principles of psychological assessment based on text information, performed the sample set data establishment and data processing of the assessment and analysis model of psychological health, conducted network establishment, training and simulation, carried out a case experiment and its result analysis, explored the cause analysis of college students' psychological health problems, and finally discussed the prevention and intervention of college students' psychological health problems. The model inputs the data processed by the sliding window into the feature extraction layer; the feature extraction is realized by alternately cascading convolutional layers and pooling layers; the convolution and pooling operations are performed on the data unit, and finally learned from various signal sources. This theory avoids the substantive question of what is the relationship between the internal characteristics of the subjects themselves and the tasks and external environments faced by the subjects. The specific implementation method is to add a partially connected convolutional layer in front of the fully connected layer of the traditional neural network. The study results show that the input and output of the CNN-based the assessment and analysis model of college students' psychological health are the college students' mental health data and assessment results, respectively, and the optimal hyper-parameters of the model are determined through fold cross-validation analysis to improve the model's over-fitting problem. After the training is completed, the model can predict the changes in college students' psychological state in the future through the psychological test data. The CNN uses supervised machine learning method to construct an assessment and analysis model of college students' psychological health, and establishes the mapping relationship between college students' personal background and their psychological health. The network error continuously adjusts network connection weight according to gradient descent algorithm to minimize its error, so that the convolutional layer and the pooling layer can learn the optimal feature representation of the input data.

## Figures and Tables

**Table 1:** Distribution of sample characteristics in four different layer of the CNN in the assessment and analysis model of psychological health of college students.

Item	Feature extraction layer	Convolutional layer	Pooling layer	Full connection layer
Text corpus	*A*	*d*	(5)	[4]
Vector model	*C*	*a*	(1)	[2]
Indicator evaluation	*C*	*b*	(2)	[1]
Cognition	*D*	*c*	(2)	[5]
Adaptation	*D*	*c*	(4)	[5]
Emotion	*B*	*a*	(3)	[5]
Pressure	*B*	*a*	(5)	[2]
Guidance	*A*	*d*	(2)	[4]
Individual growth	*D*	*c*	(1)	[3]
Positive relationship	*C*	*b*	(1)	[2]
Environment control	*A*	*a*	(5)	[1]
Experiential learning	*C*	*b*	(4)	[1]
Self-regulation	*D*	*c*	(3)	[2]
Self-acceptance	*D*	*b*	(3)	[3]
Self adaptability	*A*	*d*	(3)	[5]
Self-motivation	*A*	*b*	(2)	[4]
Self-awareness	*B*	*a*	(1)	[3]
Sensitivity	*C*	*a*	(5)	[2]
Initiative	*D*	*d*	(4)	[1]

*Note. A*-Multivariate; *B*-Multilevel; *C*-Strong coupling; *D*-Autonomy; *a*-Purposefulness; *b*-Emotional control; *c*-Independence; *d*-Interpersonal perception; (1)-Cooperation; (2)-Fulfillment; (3)-Responsibility; (4)-Purpose; (5)-Competence; [1]-Variability; [2]-Urgency; [3]-Uncertainty; [4]-Complexity; [5]-Multiple.

## Data Availability

The data used to support the findings of this study are available from the corresponding author upon request.
